# Selection of reference genes for expression analysis using RT-qPCR in the dissemination system of *Heliothis virescens* ascovirus 3 h (HvAV-3h)

**DOI:** 10.1038/s41598-017-07684-w

**Published:** 2017-08-01

**Authors:** Zi-Shu Chen, Ning-Ning Han, Jian-Hong Li, Guo-Hua Huang, Hu Wan

**Affiliations:** 1 Hubei Insect Resources Utilization and Sustainable Pest Management Key Laboratory, College of Plant Science & Technology, Huazhong Agricultural University, Wuhan, 430070 Hubei China; 2 Institute of Virology, College of Plant protection, Hunan Agricultural University, Changsha, 410128 Hunan China

## Abstract

Ascoviruses are double-stranded DNA viruses that mainly infect noctuid larvae, and are transmitted by the parasitoid wasp *Microplitis similis* Lyle. Ascovirus-parasitoids wasp-noctuid insects constitute the dissemination system. Selection of suitable reference genes for the dissemination system could play an important role in elucidating the pathogenic molecular mechanisms of ascovirus. Unfortunately, such studies on potential reference genes in the dissemination system of ascoviruses are lacking. In the present study, we evaluated 11 candidate reference genes: *β-actin1* (*ACT1*), *β-actin2* (*ACT2*), *elongation factor 1* (*EF1*), *elongation factor 2* (*EF2*), *ribosomal protein L10* (*L10*), *ribosomal protein L17A* (*L17A*), *superoxide dismutase* (*SOD*), *28S ribosome* (*28S*), *Tubulin* (*TUB*) and *18S ribosome* (*18S*). The samples were originally from various virus concentrations and points-in-time of experimental treatments using RefFinder and four algorithms. The results showed that *EF1* was the most stable internal gene in *S. exigua* and *M. similis* and that *EF2* was the most stable in the IOZCAS-Spex-II-A cell line, and the stability of reference genes were confirmed via the expression levels of two inhibitor of apoptosis-like (*iap*-like) genes from *Heliothis virescens* ascovirus 3 h (HvAV-3h). This study provides a crucial basis for future research that explores the molecular mechanisms of the pathogenesis of ascoviruses.

## Introduction

Ascoviruses (Ascoviridae) are circular double-stranded DNA viruses of insects that attack the most common lepidopteran species of the *Noctuidae*, *Crambidae* and *Plutellidae* families^1–3^. *Heliothis virescens* ascovirus 3h genome length is 190, 519 bp and is a strain of the species *Heliothis virescens* ascovirus 3a^4, 5^. HvAV-3h has strong pathogenicity to *S. exigua*, *Spodoptera litura* and *Helicoverpa armigera* ^6–8^. However, ascoviruses must be transmitted by parasitoid wasps in the field. A potential application value of ascoviruses for bio-control was predicted because of the dissemination system and acute pathogenic features of ascoviruses, which was different from other insect viruses, e.g., nucleopolyhedrovirus (NPV). Research on the pathogenic molecular mechanisms is relatively deficient for ascoviruses, and research on implementing a stabile reference gene for ascoviruses has not been performed. RT-qPCR is usually used in research on pathogenic molecular mechanisms. To achieve RNA quantitation and data normalization with different samples, we selected an optimal reference gene to correct the reliability and accuracy of quantitative results. Due to its simple operation, high sensitivity, low pollution and good repeatability, RT-qPCR is widely used in basic science research^9^. However, diverse samples have different RNA efficiencies of extraction, RNA quality, and reverse transcription efficiency of the product^10^. Therefore, it is important to ensure a high quality of RNA in RT-qPCR^11^. Generally, housekeeping genes are considered to be relatively stable expressed in all cell types and physiological states^12^. However, some studies have indicated that the expression quantity of housekeeping genes in different cell types, tissues, experimental conditions and under different physiological states is inconsistent^13, 14^. For example, *ACT* was unsuitable as a reference gene when human cell lines were infected by cytomegalovirus, human herpesvirus-6, camelpox virus, SARS coronavirus or yellow fever virus, and *TBP* and *PPI* were the most stable reference genes^15^. When *Spodoptera frugiperda* cells were infected with *Autographa californica* multiple nucleopolyhedrovirus (AcMNPV), the results indicated that *ECD* was a reliable reference gene for RT-qPCR and was better than *28S* as a reference gene for these experiments^16^. Incorporation of the *28S* reverse primer in oligo-dT-primed cDNA synthesis showed lower and less variable cycle thresholds in cells infected by viruses^17^. PPIA was set as the single, most-optimal internal reference gene for Israeli Acute Paralysis Virus (IAPV) infection experiments in *Bombus terrestris* ^18^. In various experimental settings and different tissues, *rRNA* genes were unsuitable as references gene because their transcription was significantly regulated^19^. *18S RNA* and *ACT* have been commonly employed as reference genes in Hymenoptera studies^16, 20^, Meanwhile, a suitable and stable reference gene was significant for the calibration of the qRT-PCR data.

Moreover, *iap*-like1 and *iap*-like2 in HvAV-3h were chosen as the target genes which in order to better verify the stability of the optimal internal gene predicted by the different algorithms and softwares. IAPs are a kind of widely distributed endogenous apoptosis suppressor protein, which plays an important role in inhibitor apoptosis in many species^21^. Therefore, *iap*-like1 and *iap*-like2 are probably associated with the molecular mechanism of rapid pathogenesis and chronic death in larvae. The *iap* genes are detectable in the most of the baculovirus genomes, such as AcMNPV, CpGV (*Cydia pomonella* granulovirus), OpMNPV (*Orgyia pseudotsugata* multicapsid nuclear polyhedrosis virus) and BmNPV (*Bombyx mori* nuclear polyhydrosis virus)^22, 23^. In this study, the stability of reference genes was assessed. The results could be used as internal controls in mRNA expression studies in ascovirus-infected *S. exigua* larvae, fat body cells (IOZCAS-Spex-II-A), and the parasitic wasp *M. similis*.

## Materials and Methods

### Insects, insect cell lines and viruses

The population of *S. exigua* larvae was originally collected from the vegetable fields of Huazhong Agriculture University in 2014. The insects were reared on artificial diets and maintained in a thermostatic chamber at 28–30 °C and 60–70% RH (14 L: 10D)^24^. Adults were fed with a 10% honey solution.

*Microplitis similis* samples were collected in an experimental cotton field of Hunan Agricultural University, Changsha, Hunan, China, and then reared under laboratory conditions^25^. The genders of newly emerged parasitoid adults were determined by recognizing the presence of the ovipositor under the microscope. Males and females were fed with a 30% honey solution. Each pair was provided with third-instar *S. exigua* larvae for propagation^26^.

The *S. exigua* fat body cell line (IOZCAS-Spex-II-A) was maintained at 28 °C in Grace’s Insect Medium (Sigma) supplemented with 10% fetal bovine serum. HvAV-3h, a strain of the species *Heliothis virescens* ascovirus 3a, was used in this study, and the hemolymph containing virion of HvAV-3h was collected from *S. exigua* larvae infected with HvAV-3h, as described previously^4^. The titer of hemolymph containing virion of HvAV-3h was 5.6 × 10^8^ pfu/ml, which was determined with the TCID_50_ method^27^.

### Sample collection

The third instar larvae molted after 24 h, were then injected with different concentrations of hemolymph containing the virion of HvAV-3h (10^0^-, 10^2^-, 10^4^-, 10^6^ and 10^8^-fold), and were harvested at 2, 4, 6, 8, and 10 days post-infection (p.i.)^7^. The collected samples were preserved in microcentrifuge tubes (1.5 ml) and instantaneously frozen using liquid nitrogen followed by storage at −80 °C.

The IOZCAS-Spex-II-A cell line was infected with different concentrations of sterile hemolymph containing viruses (10^2^-, 10^4^-, 10^6^ and 10^8^-fold) and harvested at 1, 3, 5, and 7 days p.i.^6^. The collected samples were preserved in microcentrifuge tubes (1.5 ml) and stored at −80 °C after being washed with PBS twice.

According to Li^26^, the female parasitoid wasp acquires the virus on the ovipositor and this can persistent for 4.1 ± 1.4 days. In the present study, different concentrations (10^0^- to 10^3^-fold) of the virus were used to infect the third-instar *S. exigua* larvae, which had molted after 24 h. Then they were exposed to female parasitoids for 24 h. All experiments were conducted in a controlled temperature and humidity environment (27 ± 2 °C, humidity 70 ± 10%, L14: D10). Determination of viruliferous vectors was conducted following Tillman^28^.

### RNA extraction and cDNA synthesis

Total RNA was isolated according to the Trizol RNA isolation kit manufacturer’s protocol. A total of 50 μl DEPC water was used to dissolve the RNA sediment. The A260/A280 ratio and A260/A230 ratio of the RNA were determined using a UV-1800 Spectrophotometer (SHIMADZU) and DU 730 nucleic acid/protein analyzer (BECKMAN COULTER). RNA samples with an A260/A280 ratio ranging from 1.8 to 2.0 and an A260/A230 ratio >2.0 were used for cDNA synthesis^29^. The first-strand complementary DNA was synthesized from 1 μg of total RNA with a PrimeScript RT reagent kit with gDNA Eraser (TaKaRa, Dalian, China) in a total volume of 20 μl. According to the manufacturer’s protocol, in the first step, 10 μl mixture was incubated for 2 min at 42 °C, and then 10 μl of master mix was added and incubated for 15 min at 37 °C and 5 s at 85 °C. The cDNA was preserved at −80 °C until further use.

### Reference gene selection and primer design

According to a previous study^30^, the PCR primer sequences of *S. exigua* and the IOZCAS- Spex-II-A cell line were used for quantification of the expression of the genes encoding *ACT1*, *ACT2*, *EF1*, *EF2*, *L10*, *L17A*, *SOD*, *TUB*, *18S* and *28S*, as shown in Table S1. The primers for *M. similis* (Table S2) were designed via Beacon Designer 8.0 software with the following settings: primer melting temperature, 60 ± 1 °C; primer GC content, 40–60%; primer length, 18–24 bp; and amplicon length, 100–200 bp. Other parameters were set by default. The lengths of the PCR-amplified specific products and not dimers of PCR products were assessed using gel electrophoresis. A 10-fold dilution series of cDNA was employed as a standard curve, and the reverse-transcription qPCR efficiency was determined for each gene and each treatment using the linear regression model^31^. According to the equation: E = (10^[−1/slope]^ −1) × 100, the corresponding qRT-PCR efficiencies (E) were calculated^32^. After detecting the efficiencies of the chosen primers, the primers that displayed a coefficient of correlation greater than 0.99 and efficiencies between 90% and 115% were selected for the next qRT-PCR (Table 1).

**Table 1:** PCR amplification efficiency of candidate reference genes.

	Gene	Length (bp)	Efficiency (%)	R^2^	Slope	y intercept
*S. exigua* and IOZCAS-Spex-II-A	ACT1	180	96.2	0.990	−3.418	43.804
	ACT2	180	99.4	0.994	−3.337	47.347
	DSP	195	90.3	0.994	−3.58	41.416
	EF1	150	95.9	0.990	−3.425	43.24
	EF2	150	98.2	0.992	−3.366	39.62
	L10	155	93.8	0.999	−3.48	41.363
	L17A	150	95.9	0.997	−3.425	38.247
	SOD	170	91.4	0.998	−3.547	43.508
	TUB	167	98.4	0.998	−3.362	41.029
	28S	133	102.1	0.993	−3.272	37.002
*M. similis*	18S	117	103.2	0.997	−3.248	27.247
	28S	122	111.3	0.997	−3.078	37.449
	ACT	136	114.4	0.995	−3.020	38.173
	EF1	117	114.5	0.998	−3.018	36.936
	TUB	192	110	0.995	−3.104	37.668
	SOD	181	93.3	0.996	−3.495	41.380

### Quantitative real-time PCR

Quantitative real-time PCR was performed using SsoFast^TM^ EvaGreen^®^ Supermix (Bio-Rad, Singapore) via a MyiQ^TM^ 2 Two Color Real-Time PCR Detection System (Bio-Rad). Each reaction was performed in a 20 μl total volume with 10 μl SsoFast^TM^ EvaGreen^®^ Supermix, 1 μl of cDNA template, 1 μl of 10 μM of each primer and 7 μl of nuclease-free water in an iQ^TM^ 96-well PCR plate (Bio-Rad). The program was set as follows: initial denaturation at 95 °C for 30 s, followed by 40 cycles of 95 °C for 5 s and 60 °C for 10 s. At the end of the reactions, a melting curve analysis from 65 °C to 95 °C was used to ensure amplified product consistency and specificity. All reactions were performed in triplicate.

### Statistical analysis

A stable level of each reference gene was statistically analyzed with four software packages: geNorm^33^, NormFinder^34^, BestKeeper^35^, delta cycle threshold (Ct) method^36^, and Online software RefFinder (freely available at: http://fulxie.0fees.us/?type = reference). Application of the geNorm, NormFinder, and BestKeeper tools was based on the Microsoft Excel program. When geNorm and NormFinder tools performed a stable analysis of the data, the cycle threshold (Ct) was converted into a linear scale (the highest relative quantity for each gene was set to 1). The geNorm algorithm calculated an expression stable value (M) for each gene and then compared the pair-wise variation Vn/Vn + 1. The gene with the lowest M value represented the most stable expression. A ratio of Vn/Vn + 1 below 0.15 indicated that the use of an additional reference gene would not significantly improve normalization^33^. NormFinder combined the interclass variance and intraclass variance to calculate a stable value. The assessment of the reference gene stability was dependent on the size of the stable value^34^. The raw data of cycle threshold (Ct) values (CP values) and PCR efficiency (E) of the reference genes were determined as the best fitted standards by BestKeeper. The cardinal principle for identification of stably expressed reference genes by Bestkeeper was that the expression levels of suitable reference genes should be highly correlated. Therefore, the correlation between each candidate gene and the index was calculated, describing the relation between the index and the contributing candidate reference gene by the highest R value, lowest SD and CV values (<1) and the P value^35^. We also used the online software RefFinder, which integrates the above-mentioned four algorithms (geNorm, Normfinder, BestKeeper, and the delta Ct method) to compare and rank the examined candidate reference genes. According to the results of RefFinder, candidate genes with the top rankings were considered to be the most stably expressed under the tested experimental conditions and thus could be selected as optimal reference genes. Every gene was sorted by the five different statistical approaches separately.

### Evaluation the stability of selected reference genes

The third instar larvae molted after 24 h and IOZCAS-Spex-II-A cells were infected with 10^2^-fold concentrations of hemolymph containing the virion of HvAV-3h, and then were harvested at 2 and 3 days p.i. The relative expression levels of inhibit apoptotic-like (*iap-*like) genes were measured using the most two stable reference genes and least two reference genes via Livak method^37^, respectively.

## Results

### Selection of candidate reference genes

To investigate the eight commonly used reference genes *ACT1*, *ACT2*, *EF1*, *EF2*, *L10*, *L17A*, *SOD*, *TUB*, and *28S* from *S. exigua* and the IOZCAS- Spex-II-A cell line and six reference genes, including *28S*, *18S*, *EF1*, *TUB*, *SOD*, and *ACT*, from *M. similis* (Table 1), we determined the correlation coefficient (R^2^) values of all candidates that varied from 0.990 to 0.998 across the cDNA diluted points and, concurrently, the PCR efficiency values of all pair-primers that varied between 90.3% and 114.5% (Table 1).

### Expression profiles of candidate reference genes

**Figure 1:**
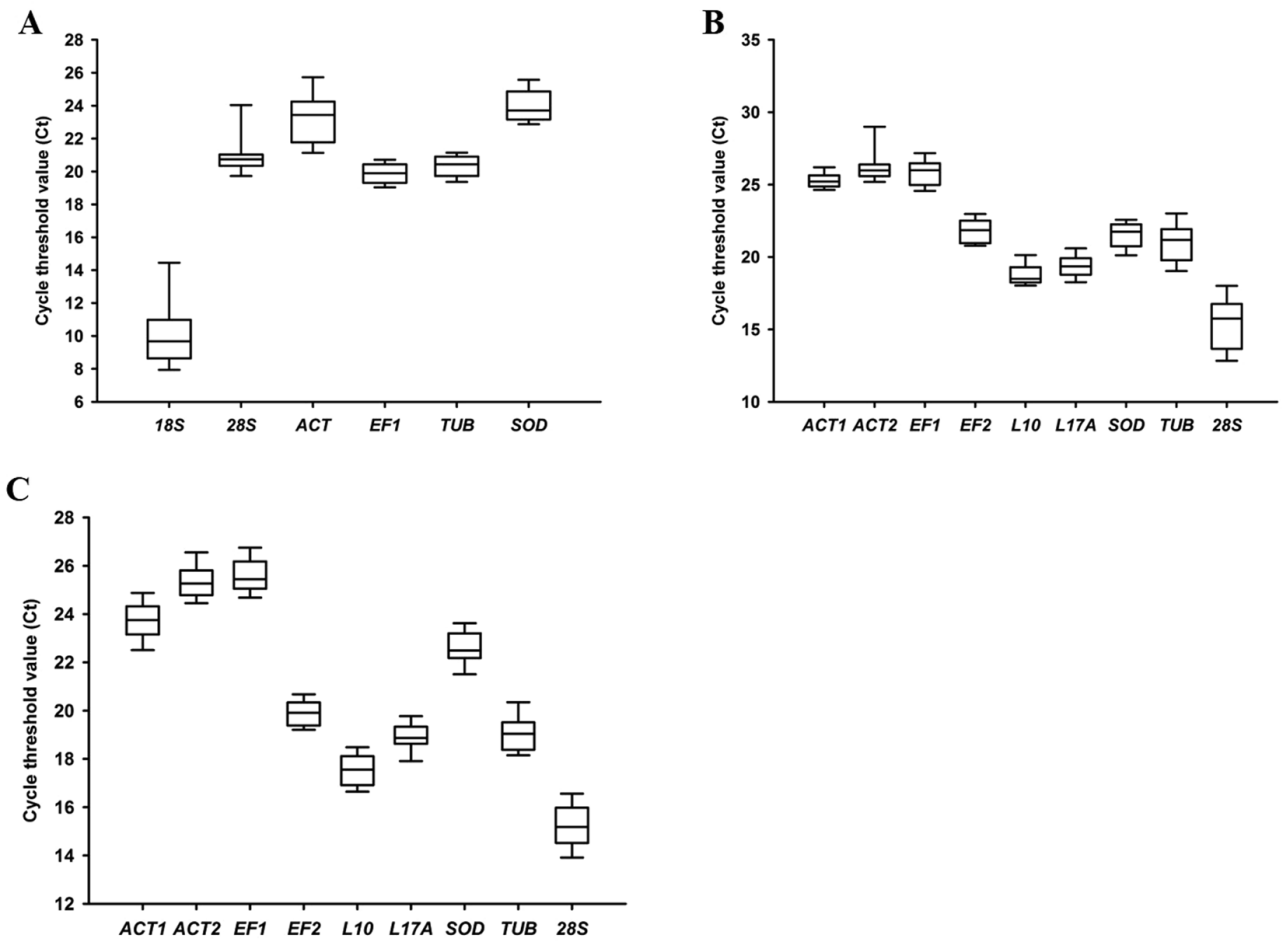
Range of Ct values in the transmission system of HvAV-3h. The above plots show expression levels of 6 candidate reference genes in (**A**) all *M. similis* samples, 9 candidate reference genes in (**B**) all *S. exigua* samples and (**C**) all IOZCAS-Spex-II-A cell line samples. Values are given as Ct values from the mean of duplicate samples. Bars indicate the standard error of the mean.

It is well known that the threshold cycle (Ct) can reflect the expression level of candidate reference genes to a certain extent. In ascovirus-infected *M. similis* (Fig. 1A), *18S RNA* with a Ct value of 7.92 had the highest expression level and was more fluctuant than the other candidate reference genes. According to the original Ct value of *S. exigua* (Fig. 1B), the highest expression reference gene was *28S* with a Ct value of 12.44, and the maximal fluctuating amplitude was 6.75. *ACT1* was the least variable compared to the other candidate reference genes. In ascoviruses-infected IOZCAS-Spex-II-A cell line samples, the Ct values of the candidate reference genes under the same threshold value for fluorescence ranged from 13.67 for *28S* to 27.07 for *EF1*, which represented the highest and lowest expression levels, respectively. The fluctuation showed no significant difference with each gene (Fig. 1C).

### Analysis of gene expression stability in ascoviruses-infected M. similis

**Figure 2:**
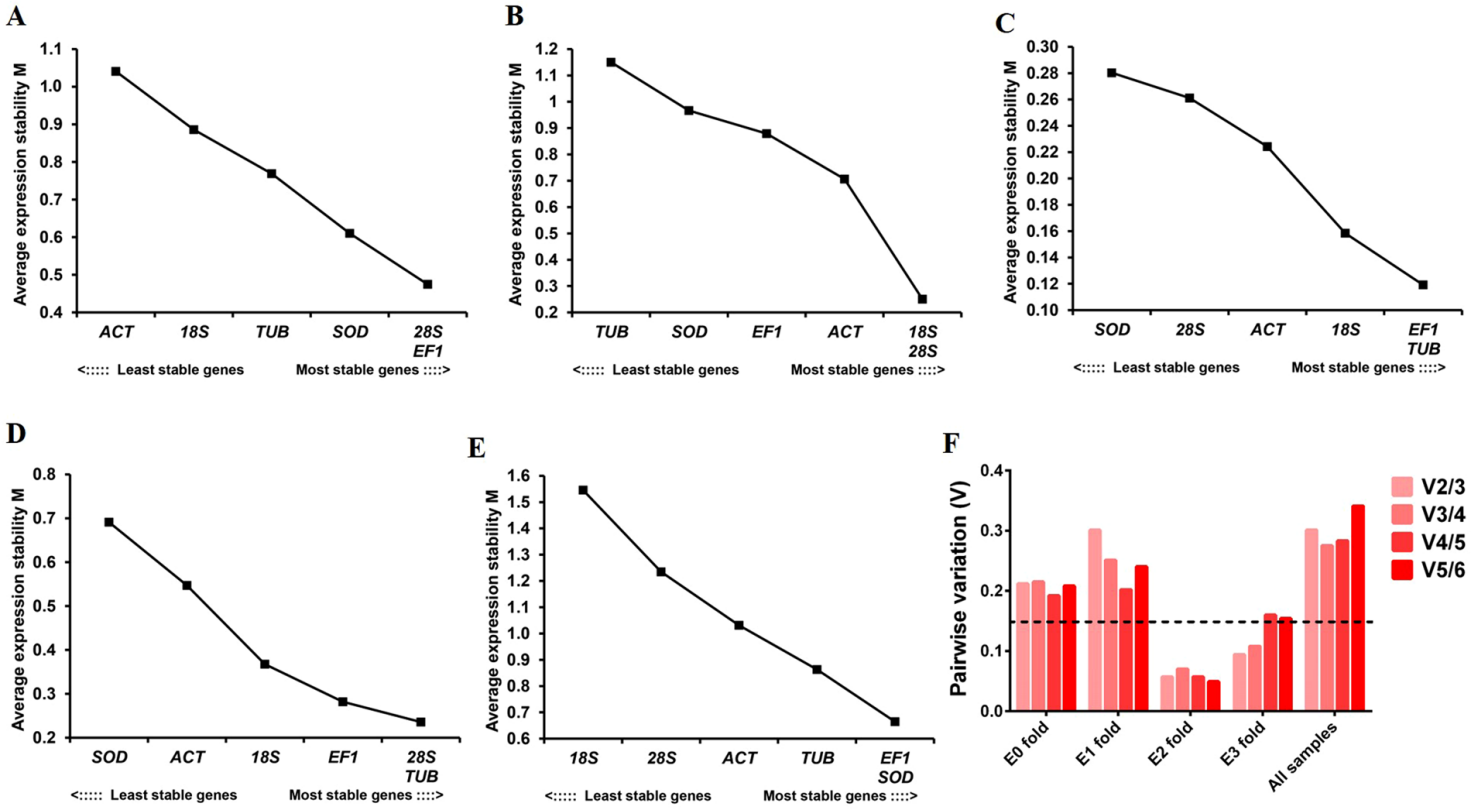
Validation of 6 candidate reference genes with these samples under ascovirus-infected *M. similis* using geNorm. Virus initial concentration (**A**), virus concentration diluted 10 multiples (**B**), virus concentration diluted 100 multiples (**C**), virus concentration diluted 1,000 multiples (**D**), and all samples set (**E**). (**A**,**B**,**C**,**D**,**E**) represent average expression stability values (M) of 6 candidate genes, and (**F**) shows the determination of the optimal number of candidate genes for normalization by geNorm analysis.

The comprehensive gene ranking of the most stable to least stable genes was *EF1*, *SOD*, *TUB*, *28S*, *ACT* and *18S*. All four programs identified *EF1* as the most stable gene in ascoviruses-infected *M. similis* samples (Table 2). Based on geNorm analysis, the four genes should not be used as reference genes for normalizing gene expression data for all samples (Fig. 2F). From the point of view of different ascovirus concentrations, except for the 10^2^-fold treatment, *EF1* was the most stable gene according to the geomean of ranking value (Table S4).

**Table 2:** Stability of candidate reference genes under ascovirus-infected conditions in *M. similis*.

Gene	Comprehensive Ranking		Delta Ct		geNorm		NormFinder		BestKeeper	
	Geomean of Ranking value	Rank	Average of SD	Rank	M value	Rank	Stability value	Rank	SD	Rank
EF1	1.00	1	1.24	1	0.66	1	0.50	1	0.56	1
SOD	1.86	2	1.29	3	0.66	1	0.68	2	0.76	3
TUB	2.91	3	1.49	2	0.86	3	0.93	4	0.57	2
28S	3.94	4	1.51	4	1.03	5	1.04	3	0.93	4
ACT	4.73	5	1.57	5	1.23	4	1.18	5	1.18	5
18S	6.00	6	2.17	6	1.54	6	2.00	6	1.66	6

### Analysis of gene expression stability in ascovirus-infected S. exigua larvae

**Figure 3:**
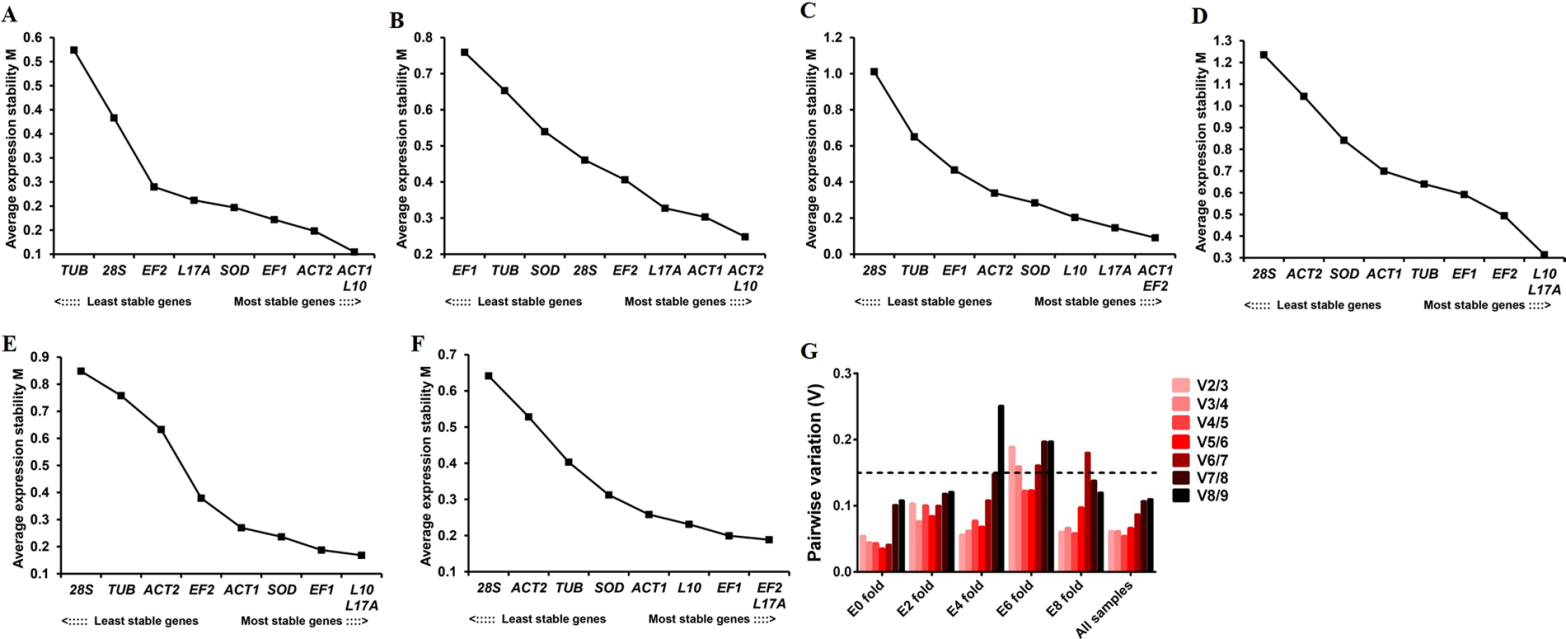
Validation of 9 candidate reference genes with these samples under different concentrations of ascoviruses in *S. exigua* using geNorm. Virus initial concentration (**A**), virus concentration diluted 100 multiples (**B**), virus concentration diluted 10,000 multiples (**C**), virus concentration diluted 1,000,000 multiples (**D**), virus concentration diluted 100,000,000 multiples (**E**), and all samples set (**F**). (**A**,**B**,**C**,**D**,**E**,**F**) represent average expression stability values (M) of 8 candidate genes, and (**G**) shows the determination of the optimal number of candidate genes for normalization by geNorm analysis.

The stability rankings generated by NormFinder were consistent with those generated by the Delta Ct method and geNorm. However, the gene stability rankings by BestKeeper analysis were different from the other three methods. In all programs except for BestKeeper, *EF1*, *L17A*, and *EF2*, showed the most stable genes (Table 3). According to the Geomean of Ranking value by Reffinder, the stability rankings from the most stable to the least stable gene in all ascovirus-infected *S. exigua* were *EF1*, *L17A*, *EF2*, *ACT1*, *L10*, *SOD*, *TUB*, *ACT2*, and *28S* (Table 3). Based on the geNorm algorithm (Fig. 3F), the gene pair *EF2*/*L17A* was the most stably expressed in all samples. Moreover, the inclusion of additional reference genes did not lower the V value below the proposed 0.15 cut-off until the ninth gene was added at 10^0^- and 10^2^-fold dilutions in all samples (Fig. 3G). From the point of different ascovirus concentrations, *L10* was the most stable gene in 10^0^- and 10^8^-fold dilutions, and *ACT2* and *EF2* were the first positions in 10^2^- and 10^4^-fold dilutions, while *L17A* was the most stable in the 10^6^-fold dilution (Table S5).

**Table 3:** Stability of candidate reference genes under ascovirus-infected conditions in *S. exigua* across all samples.

Gene	Comprehensive Ranking		Delta Ct		geNorm		NormFinder		BestKeeper	
	Geomean of Ranking value	Rank	Average of SD	Rank	M value	Rank	Stability value	Rank	SD	Rank
EF1	1.97	1	0.49	1	0.20	3	0.20	1	0.76	5
L17A	2.21	2	0.51	3	0.19	1	0.31	4	0.63	2
EF2	2.21	3	0.49	2	0.19	1	0.23	3	0.74	4
ACT1	3.81	4	0.60	6	0.26	5	0.46	7	0.52	1
L10	3.94	5	0.54	4	0.23	4	0.36	5	0.64	3
SOD	4.36	6	0.54	5	0.31	6	0.23	2	0.91	6
TUB	6.96	7	0.65	7	0.40	7	0.60	6	1.20	8
ACT2	7.74	8	0.93	8	0.52	8	0.80	8	1.06	7
28S	9.00	9	1.04	9	0.64	9	0.97	9	1.65	9

### Analysis of gene expression stability in the ascovirus-infected IOZCAS-Spex-II-A cell line

**Figure 4:**
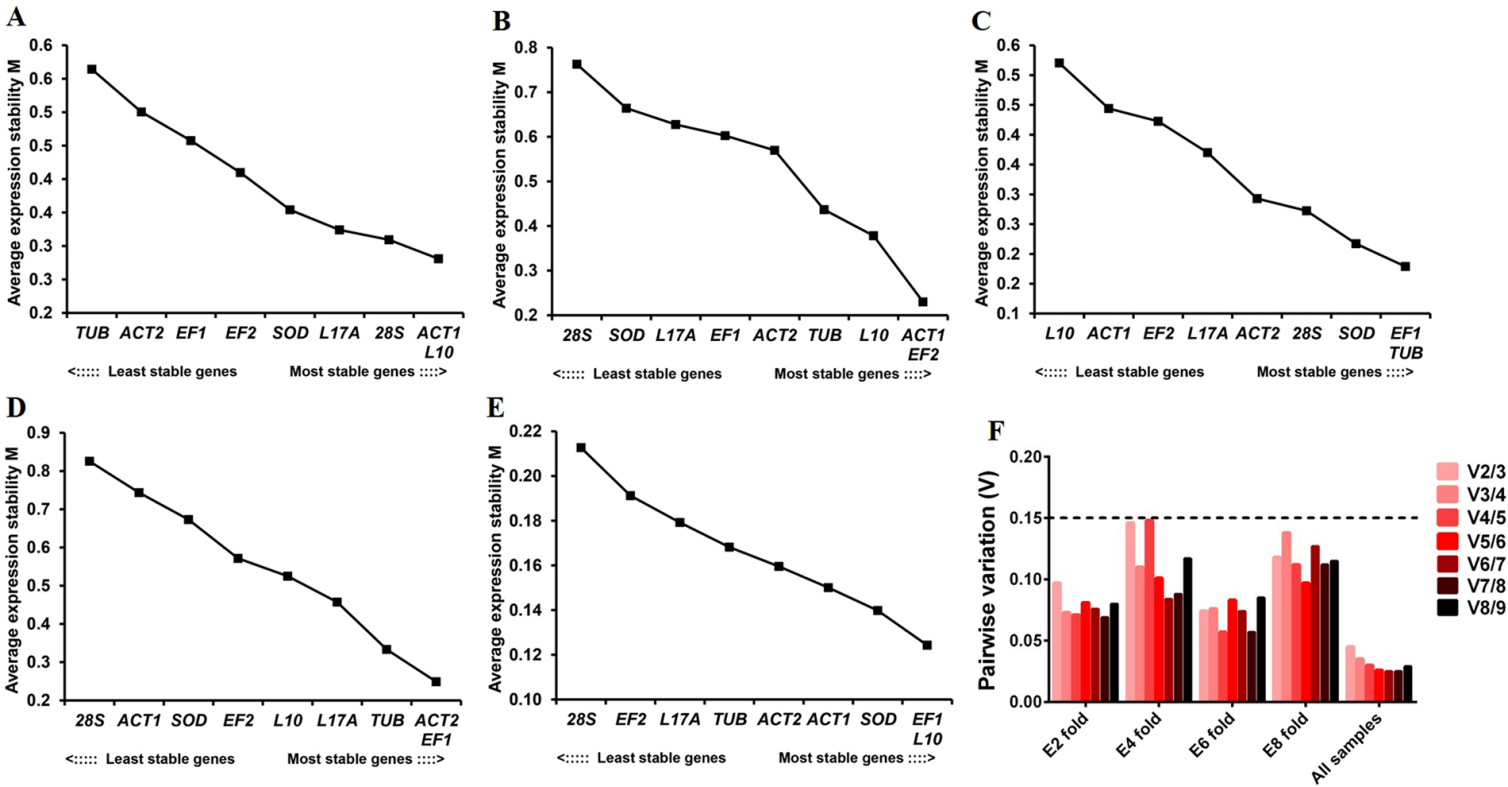
Validation of 9 candidate reference genes with these samples under different concentrations of ascoviruses in the IOZCAS-Spex-II-A cell line using geNorm. Virus concentration diluted 100 multiples (**A**), virus concentration diluted 10,000 multiples (**B**), virus concentration diluted 1,000,000 multiples (**C**), virus concentration diluted 100,000,000 multiples (**D**), and all samples set (**E**). (**A**,**B**,**C**,**D**,**E**) represent average expression stability values (M) of 9 candidate genes, and (**F**) shows determination of the optimal number of candidate genes for normalization by geNorm analysis.

The stability rankings generated by the Delta Ct method, NormFinder, and BestKeeper showed that *EF2* and *L17A* were the most stable genes, and gene stability ranked by the Delta Ct method, BestKeeper, and NormFinder were different regarding the results generated by the geNorm method (Table 4). As shown for M value and the optimal number for geNorm, all of the values were far below 1.5 (Fig. 4F). Individually, the gene pairs *ACT1*/*L10*, *ACT1*/*EF2*, *EF1*/*TUB* and *ACT1*/*EF1* were the most suitable genes in 10^2^-, 10^4^-, 10^6^- and 10^8^-fold dilutions, respectively. *EF1*/*L10* was the best pair across all samples. According to the RefFinder results, the stability rankings from the most stable to the least stable gene in the ascovirus-infected IOZCAS-Spex-II-A cell line samples were as follows: *EF2*, *L17A*, *ACT2*, *SOD*, *EF1*, *L10*, *TUB*, *ACT1* and *28S* (Table 4). As for different ascovirus concentrations, *ACT1* was the most stable gene in the 10^2^-fold dilution and *SOD* was the most stable in the 10^6^-fold dilution. *EF2* was in the first position in the 10^4^- and 10^8^-fold dilutions (Table S6).

**Table 4:** Stability of candidate reference genes under ascovirus-infected conditions in IOZCAS-Spex-II-A cells across all samples.

Gene	Comprehensive Ranking		Delta Ct		geNorm		NormFinder		BestKeeper	
	Geomean of Ranking value	Rank	Average of SD	Rank	M value	Rank	Stability value	Rank	SD	Rank
EF2	1.50	1	071	1	0.64	5	0.34	1	0.46	1
L17A	2.38	2	0.76	2	0.58	4	0.47	2	0.49	2
ACT2	2.78	3	0.78	3	0.45	1	0.52	4	0.58	5
SOD	4.28	4	0.79	4	0.70	7	0.51	3	0.58	4
EF1	4.30	5	0.80	7	0.45	1	0.55	7	0.63	7
L10	4.82	6	0.80	5	0.67	6	0.53	6	0.57	3
TUB	5.82	7	0.80	6	0.54	3	0.53	5	0.61	6
ACT1	8.00	8	0.84	8	0.71	8	0.63	8	0.66	8
28S	9.00	9	1.29	9	0.84	9	1.19	9	0.79	9

### Evaluation of selected reference genes

**Figure 5:**
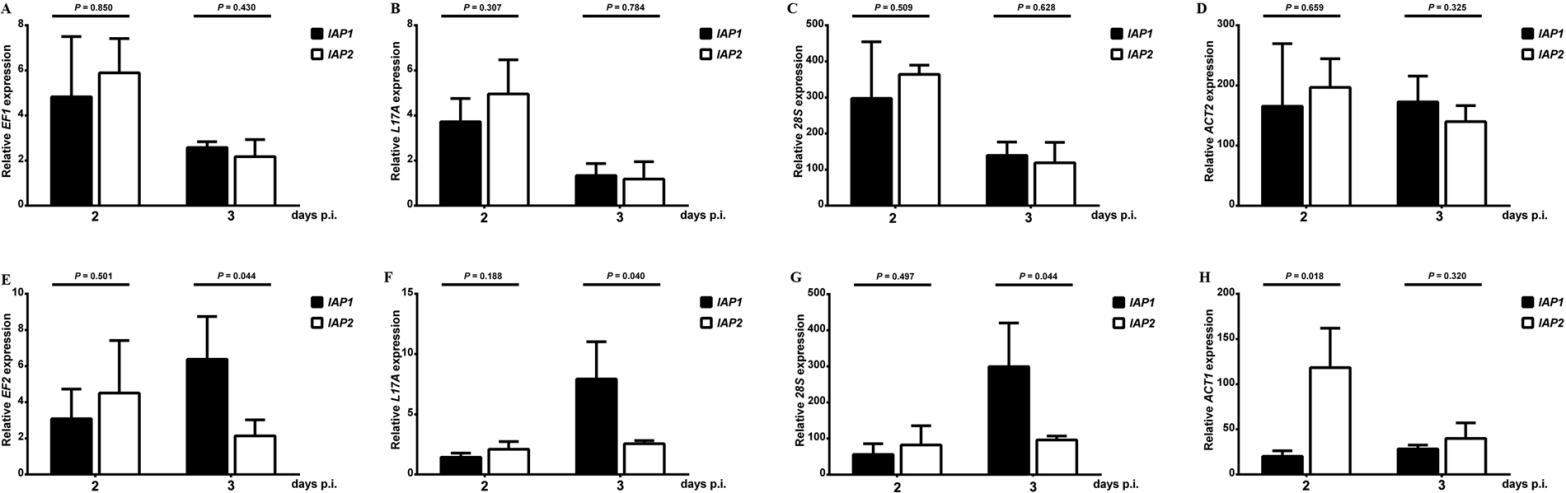
The evaluation of the selected reference genes. The relative expression of inhibitor of apoptosis-like genes normalized with the two most stable reference genes *EF1*, *L17A* (**A**,**B**) and the two least stable reference genes *28S*, *ACT2* (**C**,**D**) in *S. exigua* larvae, the two most stable reference genes *EF2*, *L17A* (**E**,**F**) and the two least stable reference genes *28S*, *ACT1* (**G**,**H**) in the IOZCAS-Spex-II-A cell line.

The results of the relative expression analysis of *iap*-like1 and *iap*-like2 (Table S3) using the two most stable reference genes *EF1* and *LA17A* in the *S. exigua* were shown in Fig. 5A,B. Additionally, *28S* and *ACT2* predicted as the two least stable genes, were applied for normalization to further verify whether the use of unstable reference gene can lead to an inaccurate relative expression (Fig. 5C,D). At the same time, the results of the relative expression analysis of *iap*-like1 and *iap*-like2 using the two most stable reference genes *EF2*, *L17A* and the two least stable reference genes *28S* and *ACT1* in the IOZCAS-Spex-II-A cell line were shown in Fig. 5E–H. In this two samples, the fold changes of the two *iap*-like genes normalized with stable reference gene showed consistent results.

## Discussion

Ascoviruses are insect-specific double-stranded circle DNA viruses that attack lepidopterans, most commonly species in the family Noctuidae^6^. HvAV-3h has been recently isolated from *S. exigua* ^4^. Li found that the early instars of *S. exigua* were significantly easier to infect with HvAV-3h compared to the later instars, using 10-fold serial dilutions (0 to 7) of HvAV-3h-containing hemolymph to infect *S. litura* larvae. There were no significant differences in larval mortalities from 10^0^- to 10^3^-fold dilutions; however, significant declines were observed at the 10^4^-fold dilution and above^7^. Compared to the healthy larval population, the typical symptoms and survival times of the diseased larval population were considerably extended, food intake was significantly reduced, and the body weight remained fairly constant in the 3^rd^ and 4^th^ instar larvae, which happened after inoculation with the ascovirus. However, the corrected mortality rates for the 1^st^ through 5^th^ instar inoculated per os were very low^8^. Therefore, the ascovirus mode of dissemination, which relies on the parasitoid wasp *M. similis*, served as a vector when the female parasitoid wasp acquired the virus. The ovipositor with the virion viability was 4.1 ± 1.4 days, and infected host larvae were still acceptable for egg laying by parasitoids. The parasitoids thereafter transmitted the virus to healthy hosts^26^. It follows that ascoviruses, with this dissemination system, have probably been regarded as significant for long-term pest control. Moreover, there are few studies that have elucidated the selection of reference genes for ascovirus infection dissemination systems. Therefore, stable expression of reference gene (s) is important for understanding the molecular mechanism of rapid pathogenesis and chronic death.

RT-qPCR is now the most sensitive method to study low-abundance mRNA from various tissue samples and experimental conditions. Thus, it is necessary to precisely determine normalization strategies^38^. Previous studies demonstrated that when reference genes were selected, the geometric mean of multiples should be used to ensure more accurate results^33^.

*Spodoptera exigua* would lead to several disordered phenomena due to the viral infection of *S. exigua*. There is no knowledge on the molecular mechanism. Thus, to gain a clear understanding of the pathogenic mechanisms, the screening of reference genes for *S. exigua* and different concentrations of ascovirus has provided a foundation for future research. At present, according to differences between physiological stages and different tissues in *S. exigua*, their suitability as reference genes was diverse with each treatment. In general, *SOD*, *ACT2*, *ACT1*, *EF1* and *GAPDH* were stably expressed in all developmental stage sample sets. *L10*, *EF2*, *L17A* and *EF1* were ranked highest in all tissue sample sets^30, 39^. The expression of these internal genes is considerably different in different experimental conditions. We therefore reassessed the stability before RT-qPCR testing. Our results indicated that *EF1* ranked hi4ghest in all sample sets by RefFinder, Delta Ct and Normfinder, whereas the BestKeeper method ranked *ACT1* as the best reference gene and *EF1* ranked at the fifth position. The subtle differences in ranking among the top order reference genes could be imputed to differences in algorithms of the employed software programs and sensitivities towards the co-regulated reference genes.

A previous study showed that after the parasitoid *M. similis* possessed the virus, the virus just stayed in the ovipositor, and the virus could only be spread in a mechanical pathway^26^. Therefore, we inferred that the parasitoid possession of the virus should barely affect the transcription factors. In most studies of Hymenoptera, *18S* or *ACT* has been commonly employed as the reference gene^40–42^. At the same time, none of the studies contributed to a comprehensive selection of internal control genes for Hymenoptera (Braconidae). In our study, although *18S* RNA had the highest expression of all the candidate reference genes, it was not the most stable. When the parasitic wasp carried the virus, the most stable reference gene was *EF1* in all sample sets by RefFinder, Delta Ct, geNorm and Normfinder. In this regard, the result was different from other studies that used *18S RNA* as an internal gene.

HvAV-3e was replicated in three noctuid cell lines from Sf9 and *Helicoverpa zea* (BCIRL-Hz-AM1 and FB33). However, HvAV-3e did not replicate in the *Pieris rapae* (Pieridae) cell line, which was non-noctuid^43^. This means that ascoviruses were likely to impact the IOZCAS-Spex-II-A cell line, which was derived from the fat body of *S. exigua*. Consequently, stable reference genes can be helpful in further research on the cytopathic effect of ascoviruses. This study showed that the IOZCAS-Spex-II-A cell line was susceptible to infection by ascoviruses. AcMNPV could also easily infect the IOZCAS-Spex-II-A cell line^16, 44^. It has been reported that with the selection of reference genes in the Sf21 cell line infected by ascoviruses, DNA-free RNA was used as a template with a combination of the Sf21 cell line *28S* gene-specific reverse primer and the oligo-dT primer for first strand cDNA synthesis. The results indicated that the Ct values were significantly higher and more variable during the course of viral infection when only the oligo-dT primer was used in the cDNA synthesis step than when the *28S*-R primer in conjunction with the oligo-dT primer was used^16^, although the stability of the reference genes was not analyzed by the geNorm, NormFinder, BestKeeper, and delta cycle threshold (Ct) method and Online software RefFinder. In our study, we applied the conventional synthesis method of cDNA using RefFinder, Delta Ct, geNorm, Normfinder and BestKeeper algorithms to analyze the Ct values. As a consequence, the results indicated that *28S* was the least suitable gene in the IOZCAS-Spex-II-A cell line across all samples, while the *EF2* gene was expressed most stably in comparison with 8 other candidate internal genes. Generally, the most stable reference gene of the IOZCAS-Spex-II-A cell line and *S. exigua* should provide the same results. We acquired *EF2* and *L17A*, which were relatively stable in screening of the reference genes for *S. exigua* and the IOZCAS-Spex -II-A cell line. However, in *S. exigua*, *EF1* was the top-ranked by RefFinder, Delta Ct and NormFinder but was in the fifth positon by BestKeeper and the third position by geNorm. This phenomenon was probably due to the virus being able to directly impact the cell line; however, virus attack of insects may be influenced by other factors such as pH values, environmental temperature and host larvae with different instars.

Pairwise variation analysis with the geNorm applet suggested the use of two or more reference genes for attaining better accuracy in normalization for most of the experimental conditions^30^. The gene pairs *EF2*/*L17A* and *EF1*/*L10* were considered the most suitable pairs of genes to normalize samples in *S. exigua* and the IOZCAS-Spex-II-A cell line, respectively, across all samples. However, conditions such as changes in *M. similis* would require normalization by three or more reference genes because the values of pairwise variations were above the cut-off range of 0.15. Thus, across all of the samples in *S. exigua* and the IOZCAS-Spex-II-A cell line, we advise the use of two reference genes under different experimental conditions.

To verify stability of candidate reference genes predicted by the RefFinder and four other algorithms, the most stable and least stable genes were applied for normalization the two *IAP* genes. Based on the sequence similarity to known IAPs, the *iap*-like genes have been chosen as the target genes. In the genomes of HvAV-3h, *IAP* genes have evolved mechanisms to reduce formation of apoptosis to guarantee the propagation of HvAV in host cells^45^. When the most stable reference genes (*EF1*, *L17A* and *EF2*) were employed to calibrated the data of gene expression in the *S. exigua* and IOZCAS-Spex-II-A cell line, the expression levels of the two *iap*-like genes revealed no significant changes. However, using the least stable reference genes *28S*, *ACT1*and *ACT2* to analyze the expression levels of the two *iap*-like genes, the results showed significant variation between two calculations which used the least stable reference genes. Therefore, it is necessary to use an appropriate stable reference gene for calibration of gene expression.

In summary, keeping in view the ecological control importance of the HvAV-3h-parasitic wasp (*M. similis*)-insect (*S. exigua*) dissemination system and pathogenic molecular mechanisms of ascoviruses, gene expression studies should continue to constitute a meaningful part of basic research with ascoviruses. Hence, establishing a best reference gene for RT-qPCR in dissemination systems will benefit researchers in this research arena. To the best of our knowledge, this is the first comprehensive report on the identification and validation of optimal candidate reference genes for accurate transcript normalization of gene expression in studies using RT-qPCR in the dissemination system of ascoviruses under various experimental conditions. We recommend the use of *EF1* in *S. exigua* and *M. similis* and *EF2* in the IOZCAS- Spex-II-A cell line. This study offers a way forward for the study of the pathogenic molecular mechanism of ascoviruses.

## Electronic supplementary material

Supplementary Information
