# Supplementary material for: Selection of reference genes for expression analysis using RT-qPCR in the dissemination system of Heliothis virescens ascovirus 3 h (HvAV-3h)
Source: Sci Rep. 2017 Aug 1;7:7045. doi: 10.1038/s41598-017-07684-w (PMC5539149; doi:10.1038/s41598-017-07684-w)
Supplement: Supplementary file 1 — Supplementary Information [file 41598_2017_7684_MOESM1_ESM.pdf]

Selection of reference genes for expression analysis using RT-qPCR in the dissemination system of *Heliothis virescens* ascovirus 3h (HvAV-3h)

Zi-Shu Chen[1,2], Ning-Ning Han[1], Jian-Hong Li[1], Guo-Hua Huang[2], Hu Wan[1*]

[1] Hubei Insect Resources Utilization and Sustainable Pest Management Key Laboratory, College of Plant Science & Technology, Huazhong Agricultural University, Wuhan 430070, Hubei, China

[2] Institute of Virology, College of Plant protection, Hunan Agricultural University, Changsha 410128, Hunan, China

Table S1. Primers used to analyze gene expression stability in ascoviruses-infected *S. exigua* and IOZCAS-spexII-A cell line.

Gene symbol	Gene name	Gene ID	Primer sequences [5'-3']
ACT1	β-actin1	AEJ38214.1	F: AAGCCTTCGATGCCACCGGGTA<br>R: TTCGGGCGTGTTTAGTGGAGGC
ACT2	β-actin2	AEJ38216.1	F: GGCTGCCGACATAGACATGCG<br>R: GGGTCCTCCACGCGGATCTT
EF1	elongation factor1	AEJ38219.1	F: TGCAGAGAAGCAAGTATTTGAGCG<br>R: CCACGAGCTTTCTCTTCCGG
EF2	elongation factor2	AAL83698.1	F: CTGACCGCGCAACCCAGACT<br>R: CACGAACATGGGGGTACCAGCG
L10	ribosomal protein L10	ABX54738.1	F: GGCTACGGTCGACGACTTCCC<br>R: GCAGCCTCATGCGGATGTGGAAC
L17A	ribosomal protein L7A	ABX55885.1	F: TGAGCTTGTCCTCTTCCTGCCC<br>R: GCTGCACGGTCGCCAGACTC
SOD	Superoxide dismutase	ABX11259.1	F: GCCGTGTGTGTTCTCAAGGGCG<br>R: GCGCCAGCTGACGTGCATCC
TUB	α-tubulin	ADL38966.1	F: CGTGACGACGTGTCTGCGGT<br>R: GCGTGAGCTCGGGTACGGTG

Table S2. Primers used to analyze gene expression stability in ascoviruses-infected *M. similis.*

Gene symbol	Gene name	Gene ID	Primer sequences [5'-3']
18S	18S ribosoma lRNAu	KJ591202	F: ATGTCTGCCTTATCAACTGTCG<br>R: TCCTTGGATGTGGTAGCCG
28S	28S ribosoma lRNAu	EU402379	F: AGTGACCACAAGGATTCTAT<br>R: GTAAACCTGTGAAACCCAAA
ACT	β-actin	XM_008557194	F: CTCTCGGTGAGGATCTTC<br>R: TTGTATTGGACTCTGGTGAT
EF1	elongation factor1	DQ538655	F: TGCTGGTACTGGTGAATT<br>R: TCCATCTTGTTGACTCCTAC
TUB	α -tubulin	JF751032.1	F: TTGACTTGACTGAGTTCCA<br>R: CCATGTATTTGCCGTGAC
SOD	Superoxide dismutase	KM244765.1	F: ATGATGGCGAACTGTGTA<br>R: AAGCATCTTCTCGTGTCA

Table S3. Primers used for evaluation of selected reference genes

Gene symbol	Gene name	Primer sequences [5'-3']
IAP-like1	Inhibition of apoptosis-like1	F: ACACCTTAGACGGAGATTC<br>R: GGATTTACAACACACCAAGT
IAP-like2	Inhibition of apoptosis-like2	F: CATCATTGTCAGCGAGAATT<br>R: GTAATGTCCGTTATCGTGTAG

Table S4. Stability of candidate reference genes under ascoviruses-infected conditions in the *M. similis*.

Gene	Comprehensive Ranking		Delta Ct		geNorm		NormFinder		BestKeeper	
	Geomean of Ranking value	Rank	Average of SD	Rank	M value	Rank	Stability value	Rank	SD	Rank
EF1(10^0-fold)	1.00	1	0.78	1	0.473	1	0.24	1	0.28	1
EF1(10^1-fold)	2.63	2	1.00	2	0.87	4	0.46	2	0.78	3
EF1(10^2-fold)	2.00	1	0.29	4	0.12	1	0.23	4	0.31	1
EF1(10^3-fold)	1.32	1	0.52	1	0.28	3	0.13	1	0.09	1
28S(10^0-fold)	1.68	2	0.94	2	0.473	1	0.61	2	0.29	2
28S(10^1-fold)	3.50	5	1.26	5	0.25	1	1.14	5	1.79	6
28S(10^2-fold)	4.16	5	0.29	5	0.26	5	0.21	3	0.52	4
28S(10^3-fold)	2.21	3	0.59	3	0.23	1	0.34	4	0.10	2
18S(10^0-fold)	4.23	5	1.06	4	0.88	5	0.75	4	0.56	4
18S(10^1-fold)	2.99	3	1.14	4	0.25	1	0.93	4	1.65	5
18S(10^2-fold)	2.06	2	0.25	1	0.16	3	0.13	2	0.44	3
18S(10^3-fold)	3.72	4	0.60	4	0.37	4	0.33	3	0.38	4
ACT(10^0-fold)	6.00	6	1.35	6	1.04	6	1.21	6	1.25	6
ACT(10^1-fold)	1.86	1	0.90	1	0.70	3	0.30	1	0.92	4
ACT(10^2-fold)	2.51	4	0.25	2	0.22	4	0.13	1	2.58	5
ACT(10^3-fold)	5.23	5	0.60	5	0.54	5	0.80	5	0.63	6
TUB(10^0-fold)	4.16	4	1.13	5	0.77	4	0.90	5	0.55	3
TUB(10^1-fold)	3.83	6	1.52	6	1.15	6	1.40	6	0.39	1
TUB(10^2-fold)	2.34	3	0.29	3	0.12	1	0.24	5	0.33	2
TUB(10^3-fold)	1.86	2	0.55	2	0.23	1	0.25	2	0.18	3
SOD(10^0-fold)	3.41	3	0.99	3	0.61	3	0.65	3	0.58	5
SOD(10^1-fold)	3.08	4	1.08	3	0.96	5	0.66	3	0.56	2
SOD(10^2-fold)	6.00	6	0.32	6	0.28	6	0.28	6	0.65	6
SOD(10^3-fold)	5.72	6	0.98	6	0.69	6	0.90	6	0.58	5

Table S5. Stability of candidate reference genes under ascoviruses-infected conditions in the *S. exigua*.

Gene	Comprehensive Ranking		Delta Ct		geNorm		NormFinder		BestKeeper	
	Geomean of Ranking value	Rank	Average of SD	Rank	M value	Rank	Stability value	Rank	SD	Rank
L10 (10^0-fold)	2.00	1	0.38	1	0.10	1	0.20	4	0.17	4
L10 (10^2-fold)	2.38	3	0.59	4	0.25	1	0.18	4	0.29	2
L10 (10^4-fold)	3.94	4	0.73	3	0.21	4	0.28	4	0.66	5
L10 (10^6-fold)	1.78	2	0.91	2	0.31	1	0.22	1	0.77	5
L10 (10^8-fold)	1.78	1	0.66	1	0.17	1	0.32	2	0.33	5
ACT1 (10^0-fold)	3.66	5	0.42	5	0.10	1	0.29	6	0.24	6
ACT1 (10^2-fold)	3.08	4	0.58	3	0.30	3	0.09	2	0.37	5
ACT1 (10^4-fold)	1.86	2	0.68	2	0.09	1	0.05	2	0.60	3
ACT1 (10^6-fold)	4.24	5	0.96	3	0.70	6	0.29	3	0.88	6
ACT1 (10^8-fold)	3.66	4	0.85	6	0.27	5	0.77	6	0.14	1
EF2 (10^0-fold)	3.03	3	0.42	6	0.24	7	0.10	2	0.10	1
EF2 (10^2-fold)	5.23	5	0.70	5	0.41	5	0.45	5	0.54	6
EF2 (10^4-fold)	1.41	1	0.68	1	0.009	1	0.05	1	0.61	4
EF2 (10^6-fold)	2.63	3	0.99	4	0.49	3	0.36	4	0.55	1
EF2 (10^8-fold)	3.22	3	0.69	3	0.38	6	0.22	1	0.71	6
EF1 (10^0-fold)	4.47	6	0.41	4	0.17	4	0.26	5	0.22	5
EF1 (10^2-fold)	9.00	9	1.13	9	0.76	9	1.08	9	0.92	9
EF1 (10^4-fold)	7.48	8	1.19	7	0.47	7	1.11	8	1.32	8
EF1 (10^6-fold)	4.36	6	1.18	5	0.59	4	0.90	6	0.64	3
EF1 (10^8-fold)	3.72	5	0.71	4	0.19	3	0.50	4	0.26	4
L17A (10^0-fold)	2.21	2	0.39	2	0.21	6	0.08	1	0.12	2
L17A (10^2-fold)	1.86	2	0.57	1	0.33	4	0.08	1	0.30	3
L17A (10^4-fold)	4.36	5	0.74	4	0.15	3	0.35	5	0.69	6
L17A (10^6-fold)	1.68	1	0.91	1	0.31	1	0.27	2	0.66	4
L17A (10^8-fold)	1.86	2	0.67	2	0.17	1	0.45	3	0.24	2
ACT2 (10^0-fold)	5.66	7	0.46	7	0.15	3	0.36	7	0.28	7
ACT2 (10^2-fold)	1.57	1	0.58	2	0.25	1	0.11	3	0.27	1
ACT2 (10^4-fold)	3.66	3	0.77	5	0.34	6	0.19	3	0.48	2
ACT2 (10^6-fold)	8.24	8	1.69	8	1.04	8	1.48	8	1.74	9
ACT2 (10^8-fold)	7.00	7	1.05	7	0.63	7	0.92	7	1.40	7
SOD (10^0-fold)	3.41	4	0.40	3	0.20	5	0.14	3	0.13	3
SOD (10^2-fold)	7.00	7	0.80	7	0.54	7	0.60	7	0.66	7
SOD (10^4-fold)	5.96	7	0.82	6	0.28	5	0.50	6	0.87	7
SOD (10^6-fold)	7.24	7	1.39	7	0.84	7	1.12	7	1.28	8
SOD (10^8-fold)	4.16	6	0.79	5	0.24	4	0.66	5	0.24	3
TUB (10^0-fold)	9.00	9	1.08	9	0.52	9	0.97	9	0.60	9

TUB (10^2-fold)	8.00	8	1.10	8	0.65	8	1.03	8	0.72	8
TUB (10^4-fold)	4.60	6	1.22	8	0.65	8	0.85	7	0.37	1
TUB (10^6-fold)	4.16	4	1.19	6	0.64	5	0.88	5	0.57	2
TUB (10^8-fold)	8.00	8	1.06	8	0.76	8	0.94	8	1.42	8
28S (10^0-fold)	8.00	8	0.82	8	0.38	8	0.72	8	0.58	8
28S (10^2-fold)	5.42	6	0.78	6	0.46	6	0.58	6	0.34	4
28S (10^4-fold)	9.00	9	2.28	9	1.01	9	2.25	9	1.33	9
28S (10^6-fold)	8.45	9	1.91	9	1.24	9	1.75	9	1.22	7
28S (10^8-fold)	9.00	9	1.17	9	0.85	9	1.06	9	1.44	9

Table S6. Stability of candidate reference genes under ascoviruses-infected conditions in the IOZCAS-spexII-A.

Gene	Comprehensive Ranking		Delta Ct		geNorm		NormFinder		BestKeeper	
	Geomean of Ranking value	Rank	Average of SD	Rank	M value	Rank	Stability value	Rank	SD	Rank
EF2 (10^2-fold)	7.20	7	0.64	8	0.46	7	0.53	8	0.46	6
EF2 (10^4-fold)	1.68	1	0.63	2	0.23	1	0.27	2	0.43	2
EF2 (10^6-fold)	5.60	7	0.50	5	0.42	7	0.33	4	0.42	7
EF2 (10^8-fold)	2.06	1	0.69	1	0.57	6	0.31	1	0.45	3
TUB (10^2-fold)	9.00	9	0.79	9	0.57	9	0.71	9	0.88	9
TUB (10^4-fold)	2.00	2	0.61	1	0.44	4	0.13	1	0.54	4
TUB (10^6-fold)	2.91	2	0.51	6	0.18	1	0.41	6	0.20	2
TUB (10^8-fold)	2.28	2	0.71	3	0.33	3	0.37	3	0.35	1
L17A (10^2-fold)	2.00	2	0.45	1	0.32	4	0.13	1	0.35	4
L17A (10^4-fold)	6.88	7	0.85	8	0.63	7	0.71	8	0.59	5
L17A (10^6-fold)	3.46	4	0.45	2	0.37	6	0.22	2	0.32	6
L17A (10^8-fold)	5.63	6	0.79	6	0.46	4	0.53	6	0.73	7
ACT1 (10^2-fold)	1.86	1	0.45	2	0.28	1	0.20	2	0.32	3
ACT1 (10^4-fold)	2.45	4	0.66	3	0.23	1	0.38	4	0.44	3
ACT1 (10^6-fold)	5.98	8	0.50	4	0.45	8	0.33	5	0.43	8
ACT1 (10^8-fold)	8.24	8	1.05	8	0.75	8	.97	8	0.83	9
L10 (10^2-fold)	2.24	3	0.53	5	0.28	1	0.39	5	0.16	1
L10 (10^4-fold)	2.45	3	0.66	4	0.38	3	0.32	3	0.18	1
L10 (10^6-fold)	9.00	9	0.79	9	0.52	9	0.76	9	0.77	9
L10 (10^8-fold)	3.16	5	0.71	2	0.53	5	0.33	2	0.63	5
EF1 (10^2-fold)	6.24	6	0.61	6	0.41	6	0.46	6	0.56	7
EF1 (10^4-fold)	6.96	8	0.80	7	0.60	6	0.67	7	0.79	8
EF1 (10^6-fold)	4.23	5	0.53	8	0.18	1	0.43	8	0.27	5
EF1 (10^8-fold)	2.38	3	0.71	4	0.25	1	0.42	4	0.42	2
SOD (10^2-fold)	4.47	5	0.52	4	0.35	5	0.33	4	0.43	5
SOD (10^4-fold)	5.89	5	0.75	5	0.66	8	0.54	5	0.60	6
SOD (10^6-fold)	1.32	1	0.42	1	0.22	3	0.19	1	0.10	1
SOD (10^8-fold)	6.74	7	0.94	7	0.67	7	0.80	7	0.70	6
ACT2 (10^2-fold)	7.48	8	0.62	7	0.50	8	0.47	7	0.68	8
ACT2 (10^4-fold)	5.96	6	0.79	6	0.57	5	0.65	6	0.73	7
ACT2 (10^6-fold)	3.41	3	0.48	3	0.30	5	0.30	3	0.20	3
ACT2 (10^8-fold)	3.16	4	0.72	5	0.25	1	0.46	5	0.45	4
28S (10^2-fold)	2.71	4	0.48	3	0.31	3	0.26	3	0.25	2
28S (10^4-fold)	9.00	9	1.11	9	0.76	9	1.04	9	0.80	9
28S (10^6-fold)	5.29	6	0.52	7	0.27	4	0.42	7	0.26	4
28S (10^8-fold)	8.74	9	1.11	9	0.83	9	1.02	9	0.80	8
